# Rapid High Yield Production of Different Glycoforms of Ebola Virus Monoclonal Antibody

**DOI:** 10.1371/journal.pone.0026040

**Published:** 2011-10-24

**Authors:** Alexandra Castilho, Natasha Bohorova, Josephine Grass, Ognian Bohorov, Larry Zeitlin, Kevin Whaley, Friedrich Altmann, Herta Steinkellner

**Affiliations:** 1 Department of Applied Genetics and Cell Biology, University of Natural Resources and Life Sciences, Vienna, Austria; 2 Mapp Biopharmaceutical, San Diego, California, United States of America; 3 Department of Chemistry, University of Natural Resources and Life Sciences, Vienna, Austria; University of Rome, Italy

## Abstract

**Background:**

Fc-glycosylation of monoclonal antibodies (mAbs) has profound implications on the Fc-mediated effector functions. Alteration of this glycosylation may affect the efficiency of an antibody. However, difficulties in the production of mAbs with homogeneous N-glycosylation profiles in sufficient amounts hamper investigations of the potential biological impact of different glycan residues.

**Methodology/Principal Findings:**

Here we set out to evaluate a transient plant viral based production system for the rapid generation of different glycoforms of a monoclonal antibody. Ebola virus mAb h-13F6 was generated using magnICON expression system in *Nicotiana benthamiana*, a plant species developed for commercial scale production of therapeutic proteins. h-13F6 was co-expressed with a series of modified mammalian enzymes involved in the processing of complex N-glycans. Using wild type (WT) plants and the glycosylation mutant ΔXTFT that synthesizes human like biantennary N-glycans with terminal N-acetylglucosamine on each branch (GnGn structures) as expression hosts we demonstrate the generation of h-13F6 complex N-glycans with (i) bisected structures, (ii) core α1,6 fucosylation and (iii) β1,4 galactosylated oligosaccharides. In addition we emphasize the significance of precise sub Golgi localization of enzymes for engineering of IgG Fc-glycosylation.

**Conclusion:**

The method described here allows the efficient generation of a series of different human-like glycoforms at large homogeneity of virtually any antibody within one week after cDNA delivery to plants. This accelerates follow up functional studies and thus may contribute to study the biological role of N-glycan residues on Fcs and maximizing the clinical efficacy of therapeutic antibodies.

## Introduction

Due to their outstanding specificities recombinant monoclonal antibodies (mAbs) have become one of the most promising products of the biopharmaceutical industry. Although the defining trait of an antibody is its specificity for the target antigen, subsequent effector functions, i.e. elimination of the antigen–antibody complex, are of major significance. These functions are mediated by the interaction of IgG Fc-domain with gamma Fc receptors (FcγRs) that are expressed by various cells. Many studies have demonstrated the critical role of the oligosaccharides attached at a single conserved site of the Fc domain for the antibody's effector functions [1], [2]. Thus glycosylation has been a focus of interest for the biopharmaceutical industry for the past several years, and cell lines have been engineered in efforts to optimize antibody products by the differential addition of fucose, galactose, bisecting N-acetylglucosamine (GlcNAc) and sialic acid [1], [2]. However, due to the large endogeneous glycosylation repertoire of the most widely used mammalian production cell lines CHO, NS0 and Sp2/0, it is currently difficult to generate mAbs with homogeneous profiles, thus hampering the investigation of the possible biological impact of specific N-glycan residues. Even the isolation of a single glycoform of a well-known glycoprotein in order to advance structure-function studies presents an enormous challenge [3]. Thus, there is an increasing demand for expression platforms that allow the rapid and efficient generation of proteins with defined homogeneous N-glycan patterns.

Recent achievements in production speed and yield of recombinant proteins has established plants as an attractive alternative expression system to cell based platforms [4], [5]. Notably, plant viral based transient expression platforms, like magnICON, allow the expression of large amounts of recombinant proteins within one week after delivery of appropriate DNA constructs [4], thus offering a new area in the field of pharmaceutical biotechnology [6]. One potential drawback of using such expression vectors is the reprogramming of the host proteome associated with the viral replication [7], [8]. These changes include alterations along the secretory pathway [9] where important posttranslational modifications, like N-glycosylation, occur. Compared to other expression platforms like yeast and bacteria, plants provide the advantage that they synthesize mammalian type complex N-glycans. Efforts towards humanization of plant glycosylation resulted in the generation of mutants that synthesize human type glycans at great uniformity [10]–[15]. Monoclonal antibodies produced in such mutants can exhibit enhanced effector function activity [10], [11], [16]. Recent advances in the development of transient expression systems have placed *N. benthamiana* in a central position; this plant species has been used at commercial scale for production of therapeutic proteins (KBP, http://www.kbpllc.com/). In the course of humanizing the plant glycosylation machinery a *N. benthamiana* glycosylation mutant ΔXTFT was generated that enables the generation of mAbs with virtually one single N-glycan species, i.e. human like biantennary N-glycans with terminal N-acetylglucosamine on each branch (GnGn structures) [13]. Such GnGn oligosaccharides provide the key structure for further elongation/modification steps, e.g. fucosylation, branching, galactosylation and sialylation. Indeed, glyco-engineered ΔXTFT served as host for the generation of recombinant proteins elongated with β1,4 galactose, sialic acid and GlcNAc branched or bisected residues [14], [15], [17], i.e. N-glycan species not naturally present in plants but frequently observed on mammalian proteins. Although these tests of concept studies demonstrate the potential of plants to be used as a versatile expression system for the generation of complex human therapeutic proteins with a customized N-glycan profile, it is not known whether these achievements translate to large scale manufacturing. Moreover, as different reporter proteins were used in these studies, limited information about the feasibility to manipulate IgG-Fc glycosylation is available.

**Figure 1:**
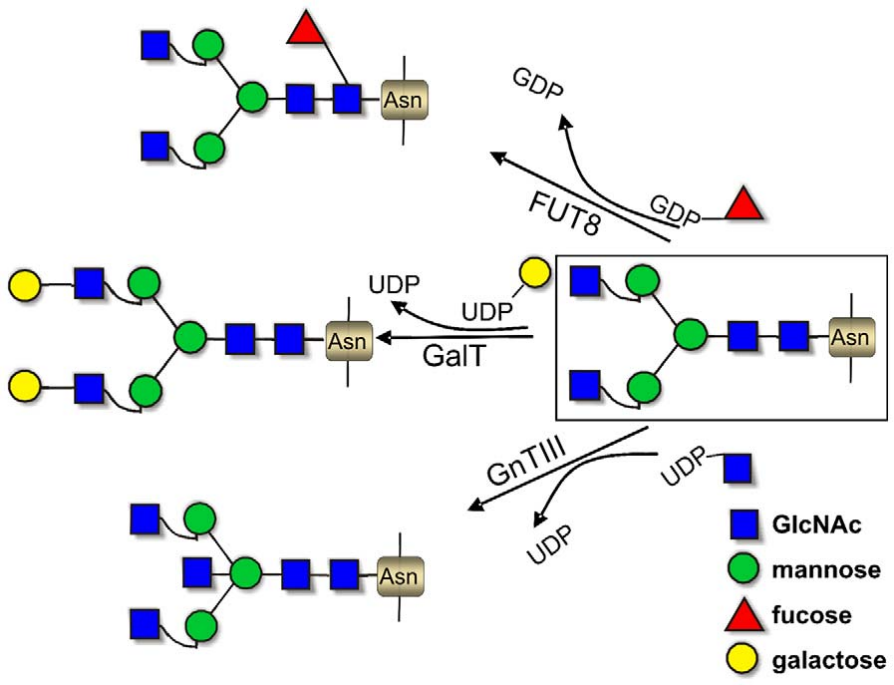
Schematic presentation of reactions catalyzed by β1,4 galactosyltransferase (GalT), N-acetylglucosaminyltransferase III (GnTIII) and core α1,6 fucosyltransferase (FUT8). GlcNAc: *N*-acetylglucosamine, UDP: Uridine diphosphate; GDP: guanosine diphosphate.

In this study we set out to evaluate, in a systematic way, the feasibility to engineer IgG Fc glycosylation upon high expression in *N. benthamiana* WT and ΔXTFT. The magnICON system which allows the expression of up to 4,8 mg mAb/gram leaf fresh weight [6] was used to generate mAbs with a customized N-glycosylation pattern avoiding time consuming transformation events. To this end we transiently co-expressed various modified human glycosylation enzymes (Figure 1) together with Ebola virus monoclonal antibody (h-13F6) [18] cloned into the magnICON system. h-13F6 was harvested at different time points and subsequently subjected to N-glycosylation analyses by ESI-MS. We demonstrate an efficient way to modify Fc glycosylation towards human glycan structures that are relatively homogenous.

## Results

### Expression of h-13F6 in *N. benthamiana* WT and ΔXTFT

**Figure 2:**
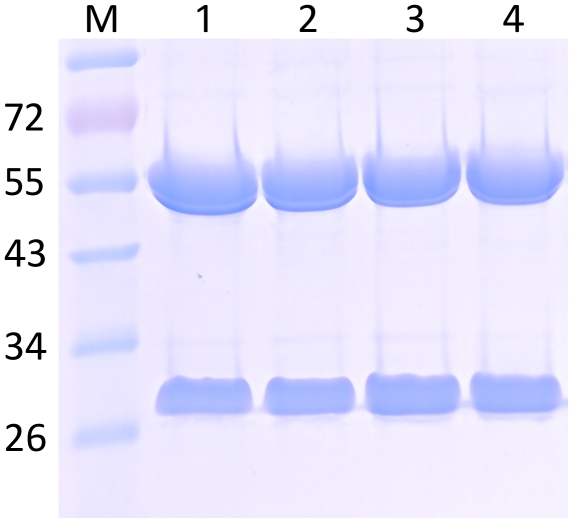
Commassie blue stained SDS-PAGE of Protein A purified h-13F6 glycoforms. Lane 1–4: h-13F6_WT_, h-13F6_ΔXTFT,_ h-13F6_ΔXTFT_ +FUT8, h-13F6_WT_+^ ST^GnTIII. M, molecular weight marker (in kDa).

**Figure 3:**
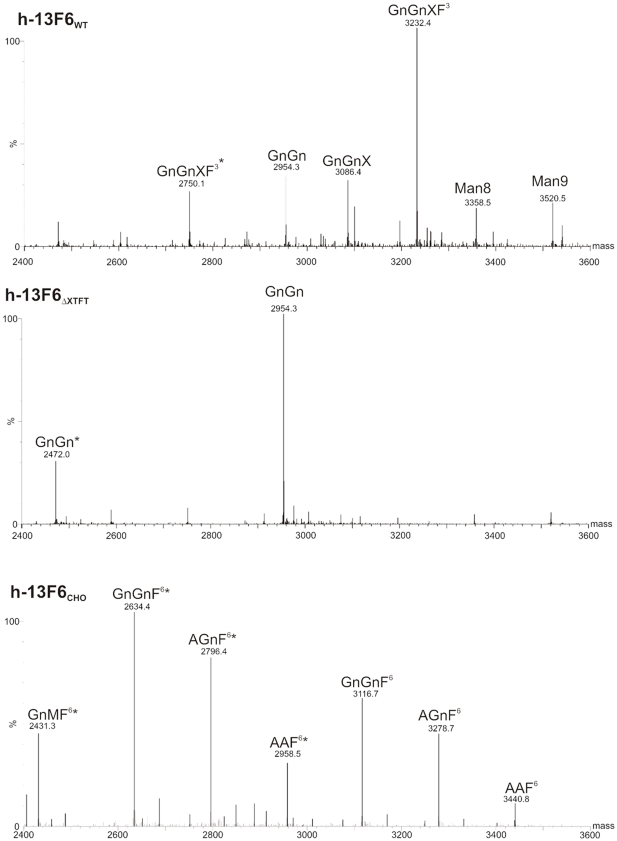
N-Glycan profiles of h-13F6 expressed in different hosts. N-glycan analysis was carried out by liquid-chromatography-electrospray ionization-mass spectrometry (LC-ESI-MS) of tryptic glycopeptides as described previously [13]; [20]. Note, that during this procedure two glycopeptides are generated that differ in 482 Da. Glycopeptide 1 is indicated with asterisks carry (*). See http://www.proglycan.com for N-glycan abbreviations. h-13F6_WT_, h-13F6_ΔXTFT_: h-13F6 generated in WT and in ΔXTFT *N. benthamiana*, respectively; h-13F6_CHO_: h-13F6 produced in CHO cells.

In this study we used the viral based magnICON system [4] for high expression of the humanized Ebola virus antibody h-13F6 [18]. Appropriate magnICON vectors carrying cDNAs from h-13F6 heavy and light chain in TMV and PVX respectively [19], were agroinfiltrated into leaves of *N. benthamiana* WT and the glycosylation mutant ΔXTFT [13]. Leaves were harvested at time points with maximal expression levels, i.e. 5–8 days post-infiltration (dpi). The expression levels were about 0.5 mg assembled IgG/g leaf biomass as estimated by Sandwich ELISA. This corresponds to about 10% of total soluble proteins. Infiltrated leaves were homogenized and extracts subjected to Protein A affinity based purification. SDS-PAGE analysis of purified h-13F6 exhibited two bands representing the heavy and the light chain, with marginal or no degradation products (Figure 2). Subsequently N-glycosylation analysis of h-13F6 was performed using liquid-chromatography electrospray ionization-mass spectrometry (LC-ESI-MS). The N-glycan profile of h-13F6 derived from *N. benthamiana* WT (h-13F6_WT_) exhibited a largely homogeneous GnGnXF^3^ pattern with plant specific β 1,2 xylose and core α1,3 fucose residues (Figure 3). Some minor glycoforms representing GnGn and GnGnX were present. h-13F6 derived from ΔXTFT (h-13F6_ΔXTFT_) carried one single dominant N-glycan species, i.e. GnGn structures (Figure 3). Both, h-13F6_WT_ and h-13F6_ΔXTFT_, exhibited only minor nonglycosylated fractions (5–10%). No significant differences in the N-glycan pattern were obtained upon harvesting at different time points (a range from 4–10 dpi was monitored). The results are in accordance with results obtained by expressing other mAbs at lower levels in the same plants [13], [17], demonstrating that high level expression of mAbs does not alter the quality of the products in terms of proteolytic degradation and Fc glycosylation. In addition the glycosylation profile of CHO (ATCC collection: CHO-K1) derived h-13F6 (13F6_CHO_) was determined and the spectrum revealed the presence of four main glycan species, all of them core α1,6 fucosylated GnM F^6^, GnGnF^6^, AGnF^6^ and AAF^6^.

In further experiments we aimed to modify the N-glycosylation profile towards human like structures by the transient coexpression of mammalian glycosyltransferases (GT) with h-13F6 in WT as well as in ΔXTFT plants. Three GTs were used to generate α1,6 fucosylated, β1,4 galactosylated and bisected oligosaccharides (FUT8, GalT, GnTIII, respectively). As the correct sub Golgi localization of the enzymes has profound consequences for the mode of modification, GTs with heterologous Golgi targeting sequences were generated.

### Generation of h-13F6 with core α1,6 fucosylated complex N-glycans

**Figure 4:**
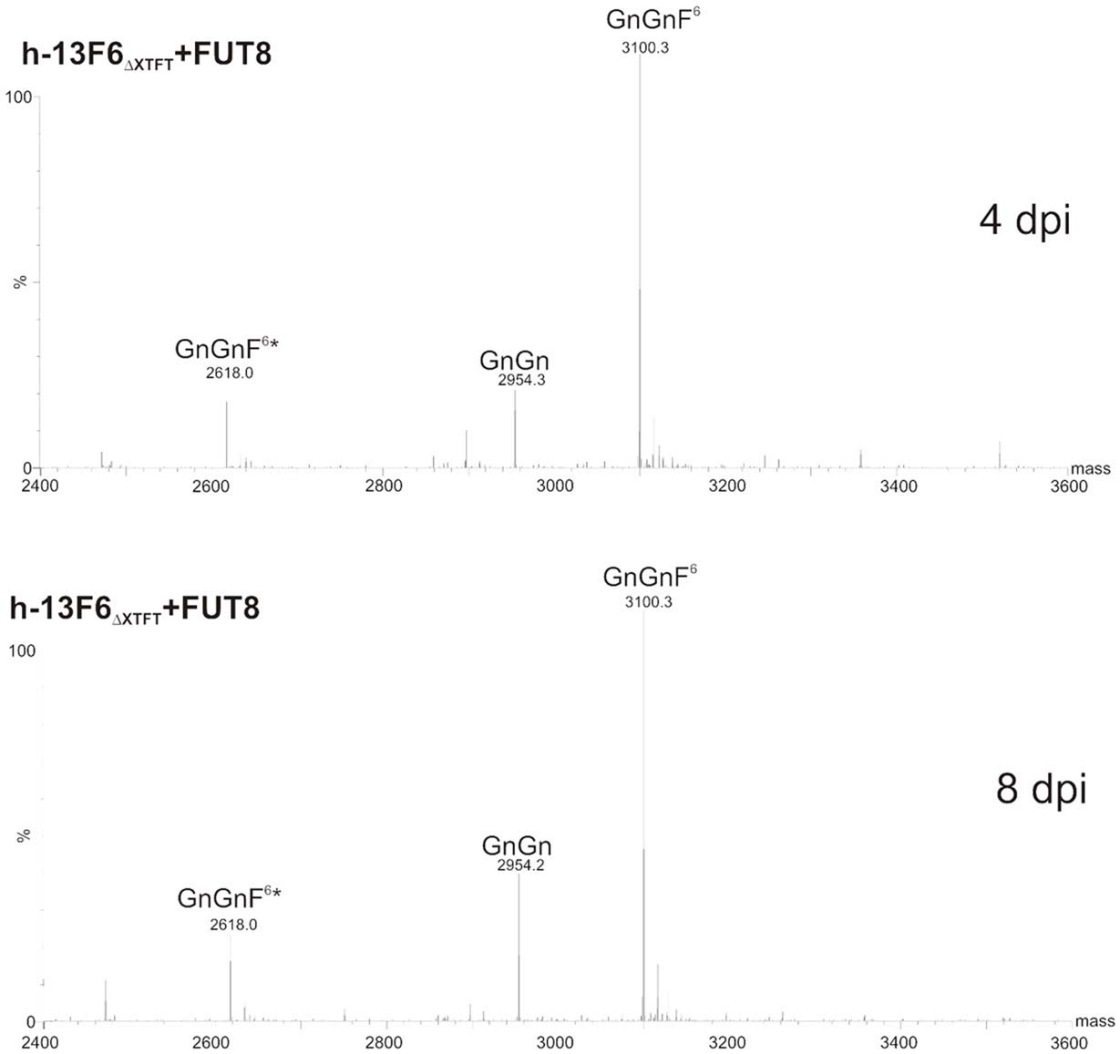
N-Glycan profiles of h-13F6_ΔXTFT_ coexpressed with human α1,6 fucosyltransferase (FUT8) and harvested at 4 and 8 dpi.

The great majority of serum IgG and mammalian cell produced mAbs carry complex N-glycans with core α1,6 fucose [20]. Though in previous studies it was demonstrated that the elimination of this sugar residue enhances Fc-mediated effector functions of many mAbs [21] the role of this abundantly present N-glycan residue has not yet been investigated in detail. Here we set out to replace the plant specific core α1,3 fucosylation by the typical mammalian type α1,6-linkage. Thus the respective human enzyme α1,6 fucosyltransferase (FUT8, Figure 1) was transiently expressed in ΔXTFT, a glycosylation mutant lacking plant specific xylose and importantly core α1,3 fucose. Agrobacteria containing FUT8 cloned into a binary vector (Figure S1) that enables low to moderate gene expression in plants [13], [17], were mixed with the magnICON based vectors containing cDNAs of h-13F6. These mixtures, containing 3 different agrobacteria strains, were infiltrated into *N. benthamiana* ΔXTFT leaves and harvested at different time points, starting with 4 dpi. As expression of h-13F6 reaches its maximum level at 5–8 dpi and then drops, the latest harvesting point was 10 dpi. Purified 13F6 subjected to LC-ESI-MS analysis shows a homogenous N-glycosylation profile with efficient core α1,6 fucosylation already at 4 dpi which remains stable until 10 dpi (Figure 4 and data not shown). Virtually all GnGn structures were converted to GnGnF^6^.

### Generation of h-13F6 with β1,4 galactosylated N-glycans

**Figure 5:**
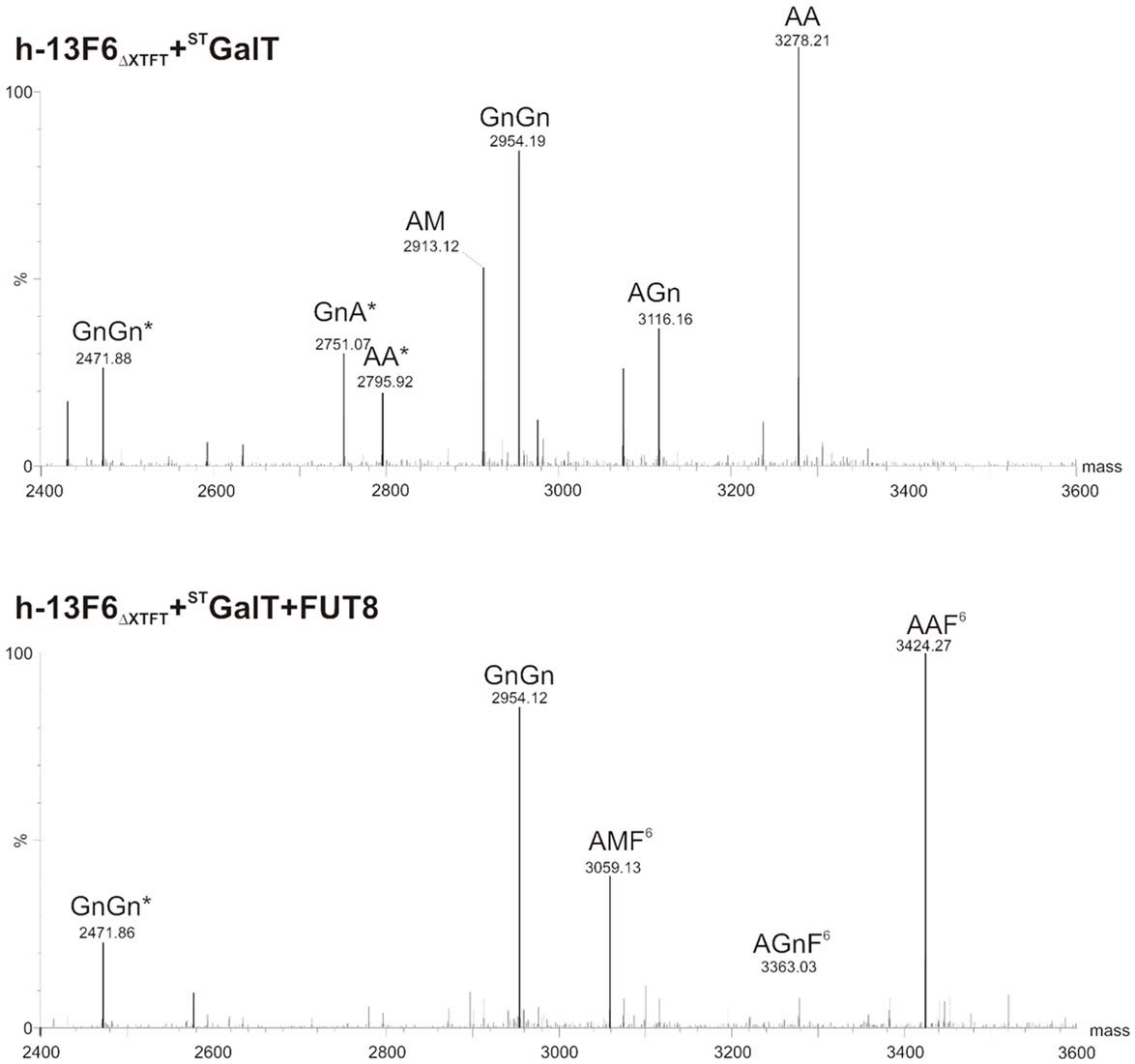
N-Glycan profiles of h-13F6_ΔXTFT_ coexpressed with (top) human β1,4 galactosyltransferase fused to the CTS of rat α2,6 sialyltransferase (^ST^GalT) and with (bottom) both^ ST^GalT and FUT8, harvested at 6 dpi.

As in other higher eukaryotes GnGn is the required acceptor substrate for further processing and elongation steps (Figure 1). In a recent study we demonstrated the efficient elongation of this structure by β1,4 galactose [17], an oligosaccharide structure frequently observed in human serum IgG and on recombinant therapeutic antibodies [2], [20], however not naturally synthesized in plants. This was achieved by the stable over-expression of a modified version of the human β1,4 galactosyltransferase (^ST^GalT) targeted to a late Golgi compartment where the final steps of glycosylation take place [17]. The catalytic domain of the enzyme was fused to the cytoplasmic-transmembrane-stem (CTS) domain of rat α2,6 sialyltransferase (ST), a potent late Golgi targeting sequence. However, the generation of such plants is time consuming and needs advanced plant transformation/regeneration knowledge which may limit their widespread use. In this study we set out to obtain efficient IgG galactosylation by the transient co-expression of the respective human enzyme in ΔXTFT (Figure 1).^ ST^GalT cloned into a binary vector (Figure S1) was mixed with h-13F6 constructs and coinfiltrated in ΔXTFT plants. Infiltrated leaves were harvested at different time points and purified mAb was subjected to N-glycosylation analyses. N-glycan profiles exhibited already at 4 dpi high fractions of galactosylated N-glycans. Biantennary N-glycans with terminal galactose on each branch (AA structures) were the major N-glycan species (Figure 5). Importantly Fc galactosylation stays consistent irrespective of the harvesting date (6 dpi and 8 dpi; Figure 5). In addition, no modified GnGn structures, monogalactosylated AGn and incompletely processed MA structures were present (Figure 5). h-13F6 harvested 10 dpi carried only minor amounts of galactosylated N-glycans (less than 5%). At this time point the major N-glycan species are non modified GnGn oligosaccharides (Figure S2). As a next step we wanted to determine whether^ ST^GalT and FUT8 can act in a synchronized way to generate galactosylated and core α1,6 fucosylated structures. Upon coexpression of both enzymes with h-13F6 in ΔXTFT GnGn structures were efficiently converted to AAF^6^ structures (Figure 5). Consistent with the results described above, galactosylation decreases 10 dpi, while fucosylation remains stable at that time point (data not shown). A surprisingly high proportion of nonfucosylated GnGn structures are present, which is in contrast to the efficient fucosylation of GnGn structures upon expression of FUT8 alone (Figure 4). It seems that the coexpression of FUT8 with ^ST^GalT inhibits the addition of α1,6 fucose to GnGn structures.

### Generation of h-13F6 with complex bisected N-glycans

**Figure 6:**
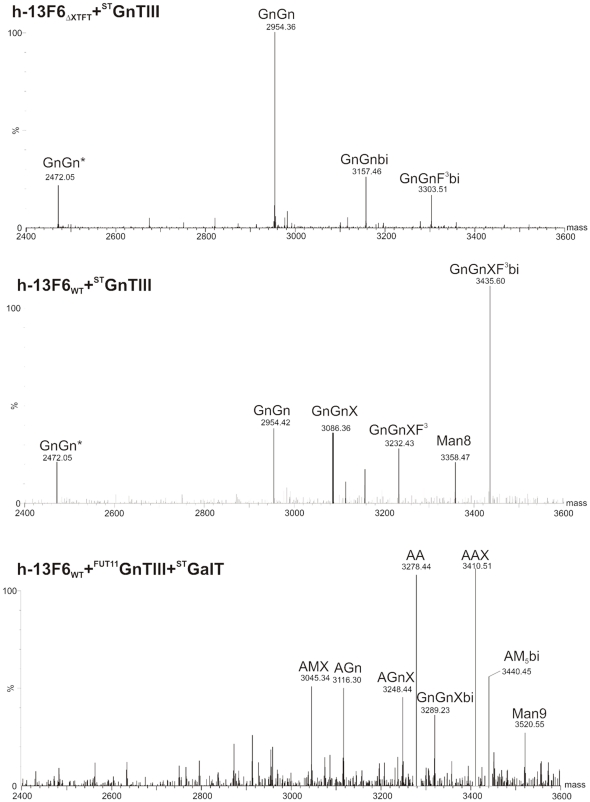
N-Glycan profiles of (top) h-13F6_ΔXTFT_ and (middle) h-13F6_WT_ coexpressed with human N-acetylglucosaminetransferase III fused to the CTS of rat α2,6 sialyltransferase (^ST^GnTIII). (bottom) N-Glycan profile of h-13F6_WT_ coexpressed with both ^ST^GalT and human N-acetylglucosaminetransferase III fused to the CTS of *Arabidopsis thaliana* core α1,3 fucosyltransferase (^FUT11^GnTIII), harvested at 6 dpi.

An additional glycoform frequently observed in human serum IgG but absent in mAbs expressed recombinantly in CHO cells, is bisecting N-acetylglucosamine [20]. To date no specific contribution to mAb or IgG activities have been assigned to these oligocaccharide structures, due in part to the difficulty to generate mAbs with this glycoform. Here we set out to generate bisecting oligosaccharides by over-expressing human N-acetylglucosaminetransferase III (GnTIII), the enzyme that adds GlcNAc to mannose in β1,4-linkage (Figure 1). Since it was shown previously that the native human enzyme expressed in tobacco generates large portions of bisecting GlcNAc, but with incompletely processed hybrid structures [22], we hypothesized that its activity interferes with the endogeneous plant N-glycosylation machinery. Indeed, a construct that targets the enzyme to a late stage in the glycosylation pathway, by fusing the catalytic domain of GnTIII to the CTS region of the rat α2,6 sialyltransferase facilitate the generation of fully processed bisecting N-glycans on erythropoietin expressed in plants [15]. This ^ST^GnTIII binary construct (Figure S1) was used for h-13F6 coinfiltration experiments in ΔXTFT. Surprisingly h-13F6 produced in this way carried unprocessed GnGn structures, only minor amounts of GnGnbi structures were detected (Figure 6). In contrast, using WT plants as expression hosts, a significant portion of bisecting structures (GnGnXF^3^bi) were generated. The quantity of GnGnXF^3^bi remains stable form 4–10 dpi (data not shown). An interesting observation was made by fusing GnTIII to a targeting sequence that directs the enzyme to a medial Golgi compartment (i.e. CTS of core α1,3 fucosyltransferase; FUT11, Figure S1). Co-expression of this modified enzyme (^FUT11^GnTIII) [15] in WT plants resulted in the generation of h-13F6 with a significant reduction of plant specific N-glycan residues, which was particularly pronounced for core α1,3 fucosylation; the three major glycoforms were GnGn and GnGnX and GnGnXF^3^ (Figure S3). Coexpression of h-13F6 with both ^FUT11^GnTIII and ^ST^GalT resulted in efficient generation of galactosylated GnGn and GnGnX structures (i.e. AAX and AA structures, Figure 6). Interestingly, no fucosylated structures were detected indicating a severe interference of the mammalian enzymes with the endogeneous glycosylation machinery.

## Discussion

The magnICON based expression system used in this study yielded reliable, uniform and high-level expression of h-13F6, a mAb being developed as a therapeutic product against Ebola virus infection [18], [19]. Here we demonstrate an efficient and rapid way to modify mAb Fc-glycosylation towards homogeneous human-like N-glycans. A series of different glycoforms of h-13F6 were generated within one week after cDNA delivery to plants. Fcs that carry quantitative amounts of β1,4 galactosylated, α1,6 fucosylated and bisecting oligosaccharides were produced. These glycoforms are abundantly present in serum IgG, but are difficult to generate individually, if at all, in established mammalian cell based expression platforms. Expression levels of all h-13F6 glycoforms were comparable with levels of about 0.5 mg assembled IgG/g leaf material. This corresponds to about 10% of total soluble protein (TSP). Previously we reported the generation of glycoengineered mAbs in the range of 0,2% of TSPs using binary vectors [13], [17]. This high expression of h-13F6 with a targeted Fc glycosylation pattern is remarkable, since it requires the synchronization of two expression systems that work in different ways. While h-13F6 is produced by viral based replicons, the glycosylation enzymes are generated by non replicating binary vectors. Our results demonstrate that viral based expression which is accompanied by a massive alteration of the entire host cell machinery does neither negatively interfere with the binary expression system nor with intracellular mechanisms facilitating efficient secretion of recombinant proteins. Efficient complex N-glycosylation, the low amount of non-glycosylated h-13F6 (5–10%) and the correct sub Golgi localization of mammalian glycosyltransferases within the Golgi compartments indicate that the secretory pathway of *N. benthamiana* is fully intact despite its massive overload of cargo protein. These results corroborate observations made with other viral based expression systems using elements from cow pea mosaic virus [23]. h-13F6 seems to be particularly stable as frequently degradation products of plant produced mAbs are observed [23], [24].

Due to the conformational complexity of IgG, Fc N-glycans are to some extent buried at the protein backbone which hinders optimal accessibility to N-glycan processing enzymes [25]. In this light efficient modification of h-13F6 oligosaccharides is amazing. Notably, a particularly highly homogeneous N-glycan profile was obtained upon infiltration of ΔXTFT with FUT8; h-13F6 Fc consisted of virtually one single N-glycan species, i.e. GnGnF^6^. Such homogeneity cannot be achieved by any mammalian based expression system and has not been reported by any other cell based expression system such as glycoengineered yeast [26]. Also, efficient galactosylation of h-13F6 was achieved by the transient co-expression of ^ST^GalT. Over 60% of the obtained structures carried terminal galactose residues. Interestingly, it seems that the coexpression of FUT8 with ^ST^GalT slightly inhibits the addition of α1,6 fucose to GnGn structures as a significant portion of h-13F6 consists of unprocessed GnGn structures. Surprisingly, h-13F6 Fc N-glycans were only marginally processed upon ^ST^GnTIII expression in ΔXTFT. On the other hand bisecting GlcNAc structures were efficiently synthesized using WT plants as expression hosts indicating the requirement of additional core N-glycan residues (in our case fucose and/or xylose) for the generation of this structure. These results are in line with those obtained recently in our laboratory by the co-expression of an erythropoietin (EPO)-Fc fusion with ^ST^GnTIII in ΔXTFT. While EPO exhibited large fractions of GnGnbi structures, only marginal amounts of bisecting structures (less than 5%) were detected on the Fc portion [15]. The results point to the importance of the protein backbone in the generation of certain glycosylation profiles and corroborate non-optimal accessibility of Fc N-glycans for some N-glycan processing enzymes. **For the readers' convenience we have listed publications derived from our laboratory in Table S1.**

While FUT8 and ^ST^GnTIII still convert respective substrates very efficiently to GnGnF^6^ and GnGnXFbi even up to 10 dpi, only minor amounts of galactosylated structures were detected at that time point upon ^ST^GalT expression. Using binary vectors, a decrease of expression of the respective proteins is expected 5–6 dpi, thus the presence of highly active ^ST^GnTIII and FUT8 10 dpi came as a surprise. It seems that fucosylation and the transfer of bisecting GlcNAc needs less enzymatic activity than galactosylation. These results are in agreement with the predominance of α1,6 fucosylated in serum IgG [20].

We demonstrate the importance of correct targeting of glycosylation enzymes within the Golgi compartments. Although upon expressing GnTIII and GalT in tobacco substantial fraction of bisecting GlcNAc and galactosylated structures were generated, large portions of unusual and incompletely processed structures were present [22], [27] indicating an interference of these enzymes with plant endogeneous glycosylation enzymes. A similar observation was made in CHO cells upon over-expression of GnTIII [21], [28]. These obscure structures were completely absent when the enzyme was targeted to a late stage of the glycosylation pathway as observed previously and in this study by the use of ST-CTS targeting signals [15], [17]. As shown for other proteins, CTS of rat α2,6 sialyltransferase seems to be a very potent late Golgi targeting sequence in plants thus targeting the human enzymes to a final stage of the glycosylation pathway. This allows endogeneous N-glycan processing to complete prior transfer of the bisecting GlcNAc residue and galactosylation. Another interesting observation is the generation of large fractions of GnGn and GnGnX structures, along with a significant reduction of core fucosylated structures by the co-expression of h-13F6 with ^FUT11^GnTIII in *N. benthamiana* WT. This reduction of fucose (and xylose) is not observed when GnTIII is targeted to a late Golgi compartment by using ST-CTS as targeting sequence. The CTS region of FUT11 targets proteins potentially to early/medial Golgi compartments. In addition, only low levels of bisecting N-glycans (less than 5%) were generated. It seems that the three enzymes, ^FUT11^GnTIII endogeneous core α1,3 FucT and XylT inhibit each other when they act in close proximity. This currently not entirely inexplicable inhibition is more pronounced for FucT. Remarkably GnGn and GnGnX structures are efficiently converted to galactosylated AA and AAX structures upon coexpression of ^FUT11^GnTIII and ^ST^GalT. Moreover, upon coexpression of these enzymes a complete inhibition of endogeneous fucosylation is obtained, indicating a severe interference of the mammalian enzymes with the endogeneous glycosylation machinery, as a consequence of intra Golgi localization. These results confirm high coordination of the glycosylation enzymes within the Golgi compartments, and point to the tremendous importance of precise targeting of the glycosylation enzymes in engineering N-glycan profiles.

The *in vivo* production of specific human-like glycoforms is in its infancy and access to truly homogeneous glycoproteins remains limited. Despite the technical hurdles associated with the introduction of human-type glycosylation machinery into a foreign organism, our results indicate that plants have the potential to offer a significant alternative to mammalian cell culture as a source of humanized glycoproteins. The clinical success of monoclonal antibodies has been demonstrated by a number of antibody therapeutics. Nevertheless, it is still very important to optimize their clinical efficacy. Clinical trials using therapeutic antibodies fully lacking core fucose residues in the Fc oligosaccharides are currently underway, and their remarkable physiological activities *in vivo* have attracted attention as next-generation therapeutic antibody approaches with improved efficacy [29], [30]. However, the biological impact of other major glycoforms present in serum IgG is largely unknown. The rapid generation of different glycoforms of virtually any mAb as described here will advance functional studies and thus significantly contributes to optimize the clinical efficacy of therapeutic antibodies.

## Materials and Methods

### Plant expression binary vectors

Binary vectors used in this investigation for the expression of mammalian glycosyltransferases in plants were previously described: ^ST^GalT [17];^ FUT11^GnTIII and ^ST^GnTIII [15]. Schematic presentation of all constructs, including FUT8, is shown in Figure S1. Generation of magnICON vectors carrying the cDNA of h-13F6 heavy and light chains is described elsewhere [19].

### h-13F6 infiltration and purification

*Nicotiana benthamiana* WT and ΔXT/FT plants [13] were grown in a growth chamber at 22°C with a16 h light/ 8 h dark photoperiod. Leaves of four to five week old plants were used for agroinfiltration experiments. Agrobacteria transformed with the TMV and PVX based-vectors containing the h-13F6 cDNA for the heavy and light chains were grown at 29°C for 24 hours. After harvesting by gentle centrifugation (5 min at 3000 g), the bacteria were ressuspended in buffer (10 mM MES pH 5.6; 10 mM MgSO_4_), mixed and diluted to a final OD_600_ of approximately 0.1–0.2. In experiments aimed to modulate plant glycosylation, agrobacteria transformed with binary vectors containing the cDNA of specific mammalian glycosyltransferases were prepared the same way and included in the infiltration mixture to be co-infiltrated with the viral-based vectors.

Approximately 200–250 mg of infiltrated leaf material was homogenized, mixed with 600 µL extraction buffer (500 mM NaCl; 100 mM Tris/HCl; 40 mM L(+) ascorbic acid; 1 mM EDTA; pH = 6,8) and incubated on ice for 10 min. After centrifugation (3×15 min at 9.600 g) supernatant was incubated with 15–20 µL ProteinA Sepharose™ Fast Flow (GE Healthcare) for 1 h30 min at 4°C. After a brief spin down, the supernatant was discarded and the Sepharose was washed 3 times with 1x PBS using Micro Bio-Spin chromatography columns (Bio-Rad). 30 µL of 100 mM Glycine/HCl pH = 2.5 were applied to the column and the eluate containing the purified IgG was neutralized by adding 1.2 µL of 0.5 M Tris.

### N-glycosylation analyses

N-glycan analyses were carried out by liquid-chromatography electrospray ionization-mass spectrometry (LC-ESI-MS) of tryptic glycopeptides as recently described [20]. Briefly, the heavy chain band of purified, SDS-PAGE separated IgGs was cut from the gel, S-alkylated, digested with trypsin, eluted from the gel fragment with 50% acetonitril and separated on a Biobasic C18 column (150×0.32 mm, Thermo Electron) with a gradient of 1–80% acetonitrile containing 65 mM ammonium formate pH 3.0. Positive ions were detected with a Q-TOF Ultima Global mass spectrometer (Waters, Milford, MA, USA). Summed and deconvoluted spectra of the glycopeptides elution range were used for identification of glycoforms. This method generates two glycopeptides that differ by 482 kDa (glycopeptide 1, EEQFNSTYR; glycopeptide 2 TKPREEQFNSTYR). Please note that the original h-13F6 produced in CHO (CHO-K1 ATCC® Catalog No. CCL-61) generates two glycopeptides that differ, compared to the plant produced version, in one amino acid: (glycopeptide 1, EEQ**Y**NSTYR; glycopeptide 2 TKPREEQ**Y**NSTYR). This change results in a mass shift of 16 Da between the two versions in LC-ESI-MS analyses. The transfer of fucose in α1,6 linkage was further investigated by PNGaseF digestion of an Fc fragment (Figure S4) that was coexpressed with FUT8. PNGaseF is a well known endoglycosidase that digests N-linked oligosaccharide, except those carrying fucose in α1,3 linkage [31]. Complete deglycosylation of the glycopeptides, including the assigned GnGnF peak, further confirms α1,6 linkage of fucose.

## Supporting Information

Figure S1 **Schematic representation of the different binary expression vectors used in this study. ^ST^GalT**: cytoplasmic tail-transmembrane-stem region CTS of rat α2,6-sialyltransferase (ST) fused to the catalytic domain of human β1,4-galactosyltransferase. **FUT8:** human core α1,6-fucosyltransferase full length; **^ST^GnTIII:** ST-CTS region fused to catalytic domain of human β1,4-mannosyl-β1,4-N-acetylglucosaminyltransferase (GnTIII); **^FUT11^GnTIII**: CTS of *A. thaliana* core α1,3-fucosyltransferase fused to catalytic domain of GnTIII. Pnos: nopaline synthase gene promoter; Tnos: nopaline synthase gene terminator; P35S: promoter of the 35S transcript of the Cauliflower Mosaic Virus; g7T: Agrobacterium gene 7 terminator; KanR: neomycin phosphotransferase II; LB: left border; RB: right border.
(TIF)

Figure S2 **N-Glycan profiles of h-13F6_ΔXTFT_ coexpressed with human β1,4 galactosyltransferase fused to the CTS of rat α2,6 sialyltransferase (^ST^GalT) harvested 10 dpi.**
(TIF)

Figure S3 **N-glycosylation profile of h-13F6 expressed in** ***N. benthamiana*** **WT together with ^FUT11^GnTIII.**
(TIF)

Figure S4 **Determination of α1,6 linkage of fucose on Fc. N-glycosylation profile of Fc coexpressed with FUT8 (A), and subsequent PNGaseF treatment (B).** While several, and particularly fucosylated GnGn glycoforms were present in **A,** upon PNGaseF only de- and nonglycosylated peptides were detected in **B**. The digestion of virtually all oligosaccharides including GnGnF further confirms α1,6 linkage of this N-glycans residue.
(TIF)

Table S1 **For convenience of readers a list of previous own publication is provided.**
(DOC)
