# Supplementary figures and images for: Rapid High Yield Production of Different Glycoforms of Ebola Virus Monoclonal Antibody
Source: PLoS One. 2011 Oct 24;6(10):e26040. doi: 10.1371/journal.pone.0026040 (PMC3200319; doi:10.1371/journal.pone.0026040)

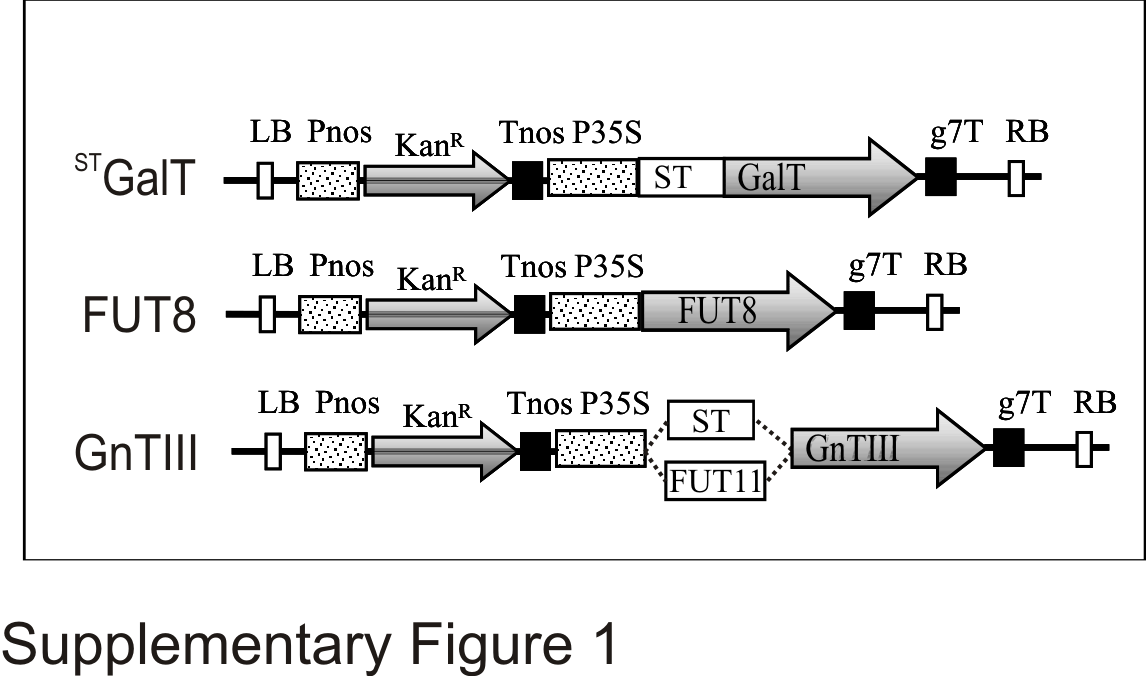

Supplement: Figure S1 — Schematic representation of the different binary expression vectors used in this study. STGalT: cytoplasmic tail-transmembrane-stem region CTS of rat α2,6-sialyltransferase (ST) fused to the catalytic domain of human β1,4-galactosyltransferase. FUT8: human core α1,6-fucosyltransferase full length; STGnTIII: ST-CTS region fused to catalytic domain of human β1,4-mannosyl-β1,4-N-acetylglucosaminyltransferase (GnTIII); FUT11GnTIII: CTS of A. thaliana core α1,3-fucosyltransferase fused to catalytic domain of GnTIII. Pnos: nopaline synthase gene promoter; Tnos: nopaline synthase gene terminator; P35S: promoter of the 35S transcript of the Cauliflower Mosaic Virus; g7T: Agrobacterium gene 7 terminator; KanR: neomycin phosphotransferase II; LB: left border; RB: right border. (TIF) [file pone.0026040.s001.tif]

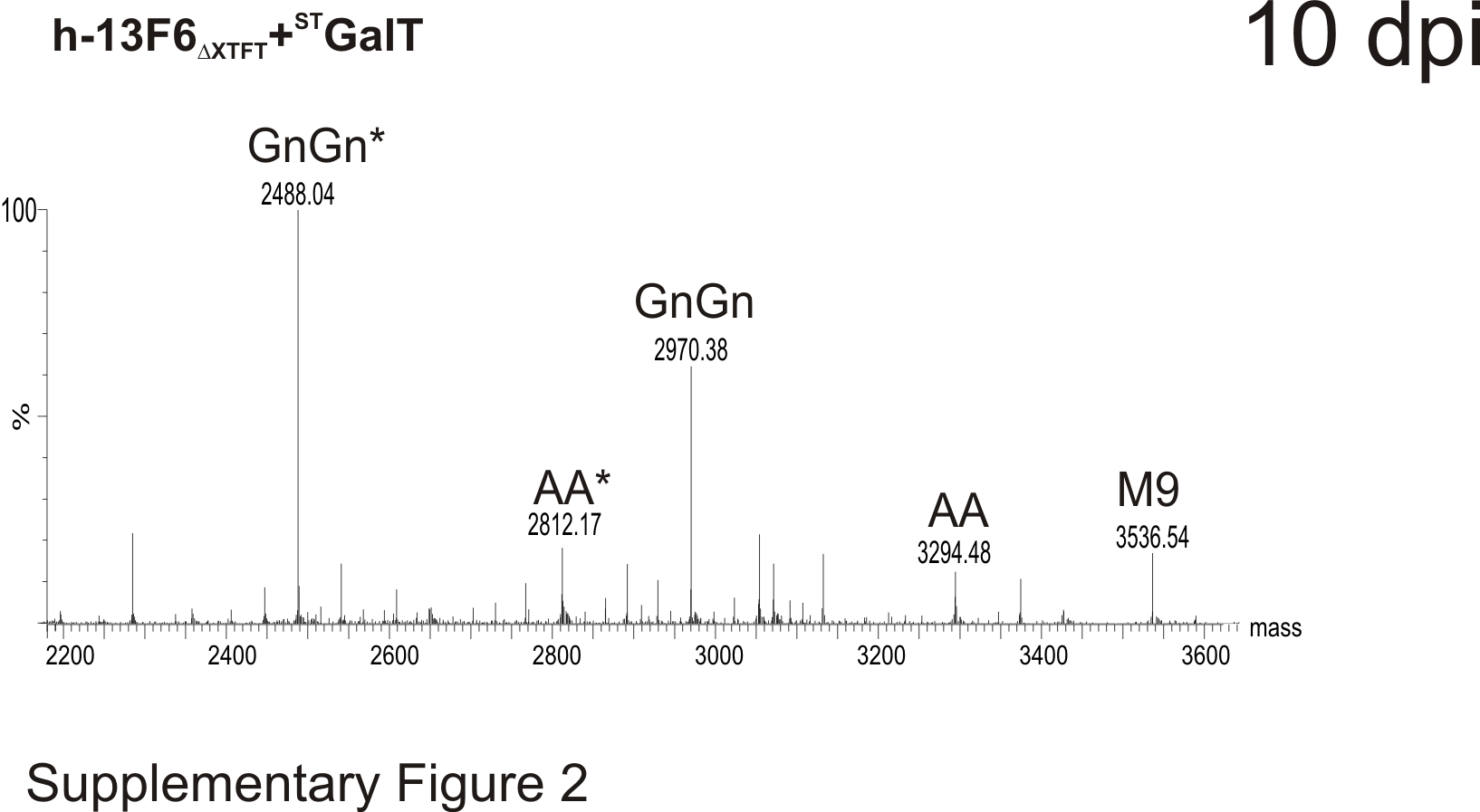

Supplement: Figure S2 — N-Glycan profiles of h-13F6ΔXTFT coexpressed with human β1,4 galactosyltransferase fused to the CTS of rat α2,6 sialyltransferase (STGalT) harvested 10 dpi. (TIF) [file pone.0026040.s002.tif]

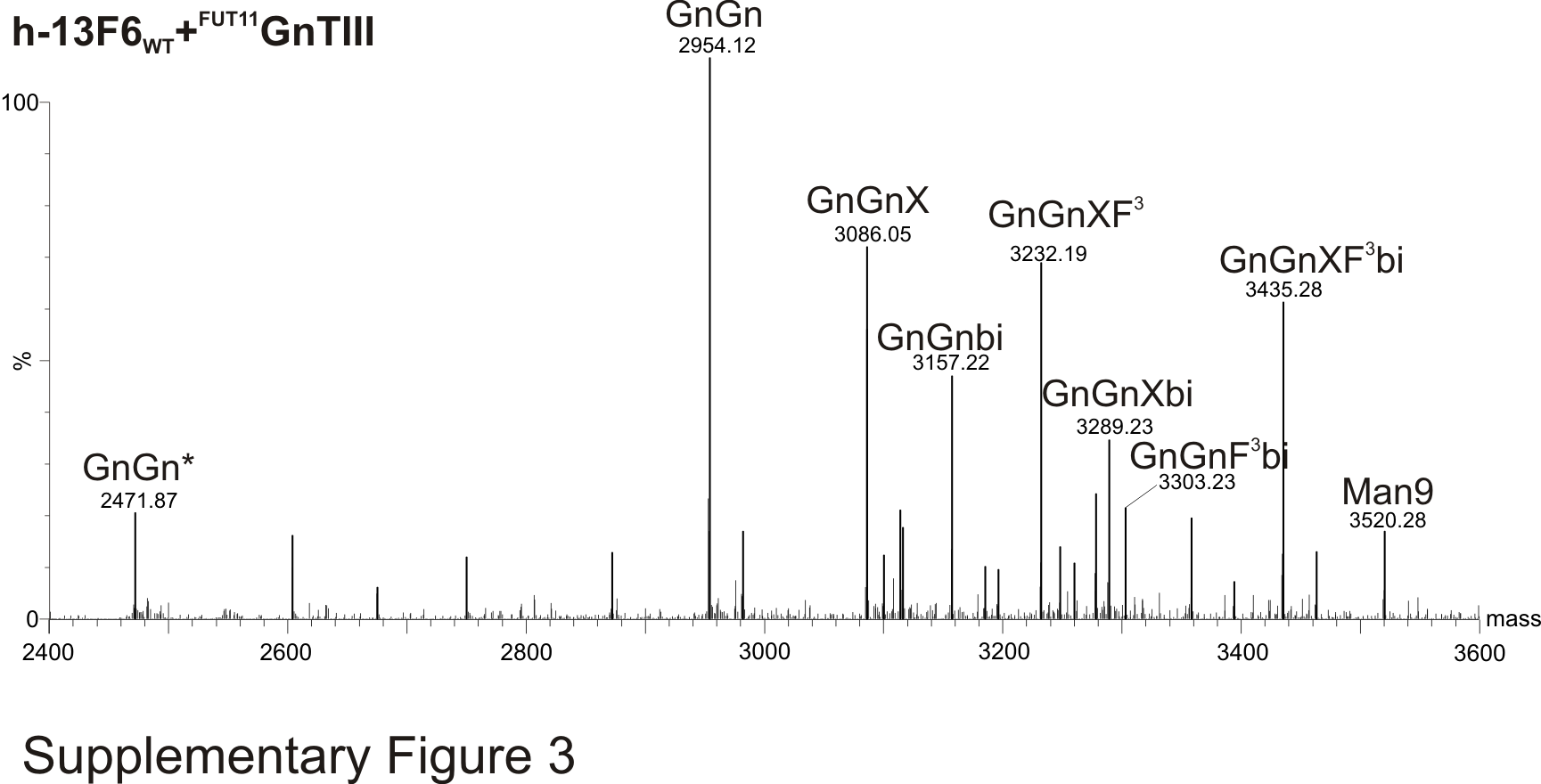

Supplement: Figure S3 — N-glycosylation profile of h-13F6 expressed in N. benthamiana WT together with FUT11GnTIII. (TIF) [file pone.0026040.s003.tif]

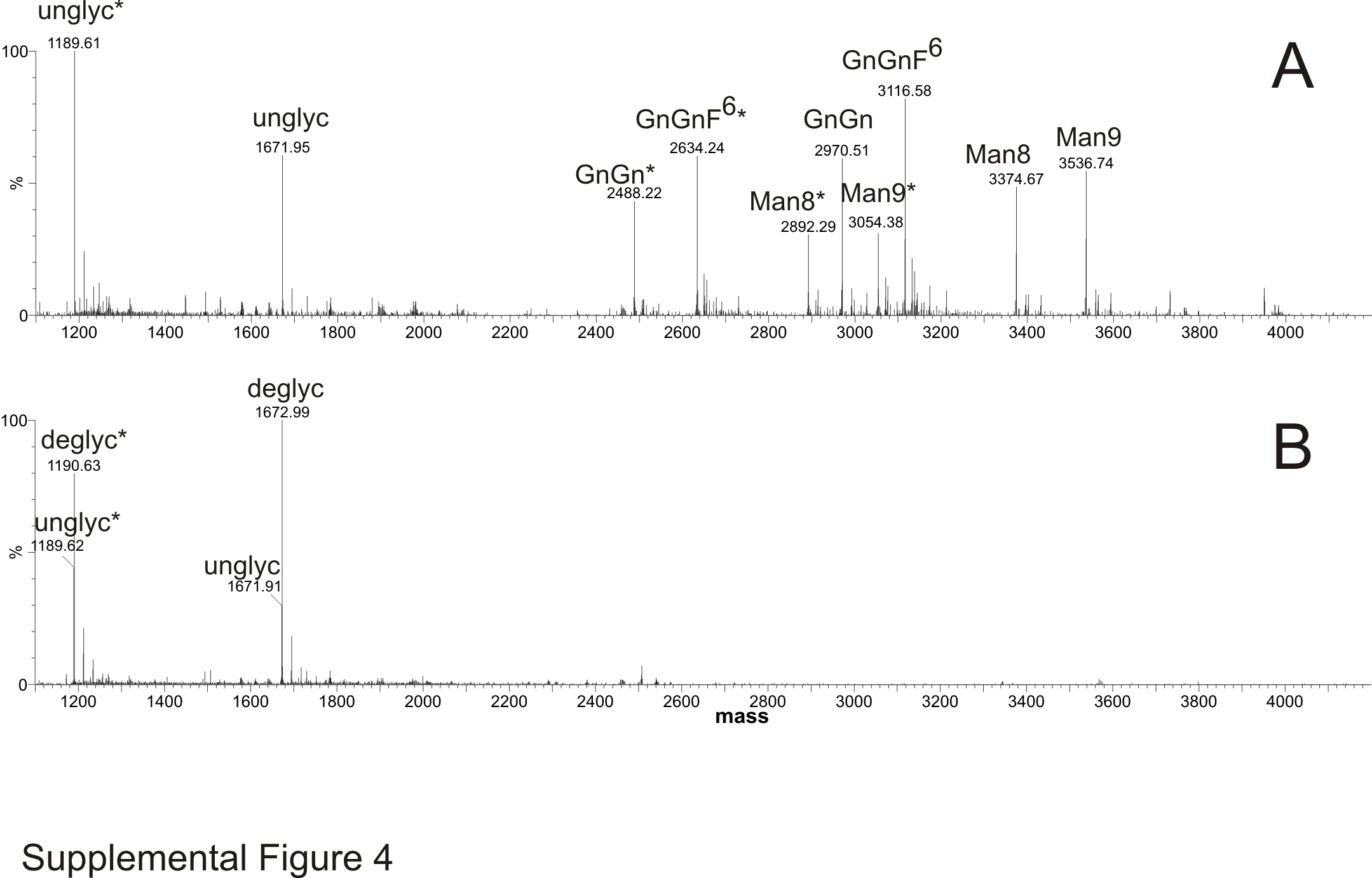

Supplement: Figure S4 — Determination of α1,6 linkage of fucose on Fc. N-glycosylation profile of Fc coexpressed with FUT8 (A), and subsequent PNGaseF treatment (B). While several, and particularly fucosylated GnGn glycoforms were present in A, upon PNGaseF only de- and nonglycosylated peptides were detected in B. The digestion of virtually all oligosaccharides including GnGnF further confirms α1,6 linkage of this N-glycans residue. (TIF) [file pone.0026040.s004.tif]
