# Supplementary material for: Rapid High Yield Production of Different Glycoforms of Ebola Virus Monoclonal Antibody
Source: PLoS One. 2011 Oct 24;6(10):e26040. doi: 10.1371/journal.pone.0026040 (PMC3200319; doi:10.1371/journal.pone.0026040)
Supplement: Table S1 — For convenience of readers a list of previous own publication is provided. (DOC) [file pone.0026040.s005.doc]

| **Previous own Publications** | **Date** | **Content** |
| --- | --- | --- |
| Schähs M, Strasser R, Stadlmann J, Kunert R, Rademacher T, Steinkellner H. Production of a monoclonal antibody in plants with a humanized N-glycosylation pattern. Plant Biotechnol J. 5:657-63. | 2007 | *A. thaliana* knock out mutants allowed the generation of mAb lacking xylose and core α 1,3 fucose N-glycan residues. |
| Strasser R, Stadlmann J, Schähs M, Stiegler G, Quendler H et al. Generation of glyco-engineered *Nicotiana benthamiana* for the production of monoclonal antibodies with a homogeneous human-like N-glycan structure.Plant Biotechnol J. 6:392-402. | 2008 | A RNAi line (ΔXTFT) that lacks plant specific glycan residues from the potential production host *Nicotiana benthamiana* was generated. |
| Stadlmann J, Pabst M, Kolarich D, Kunert R, Altmann F Analysis of immunoglobulin glycosylation by LC-ESI-MS of glycopeptides and oligosaccharides. Proteomics 8:2858-71. | 2008 | An advanced method for the rapid and accurate estimation of glycopeptides and oligosaccharides. The glycosylation profile of a series of (commercial) mAbs were compared |
| Strasser R, Castilho A, Stadlmann J, Kunert R, Quendler H et al. Improved virus neutralization by plant-produced anti-HIV antibodies with a homogeneous beta1,4-galactosylated N-glycan profile. J Biol Chem 284:20479-85. | 2009 | The generation of *in planta* terminally galactosylated mAbs is shown. This was achieved either by the stable expression of the respective human enzyme GalT in *N. benthamiana* and by its transient expression. HIV antibody served as model mAb. The importance of sub Golgi targeting of glycosyltransferases is shown. |
| Forthal DN, Gach JS, Landucci G, Jez J, Strasser R et al. (2010) Fc-glycosylation influences Fcγ receptor binding and cell-mediated anti-HIV activity of monoclonal antibody 2G12. J Immunol.185:6876-82. | 2010 | This work compares the *in vitro* and *in vivo* functional activities of different glycoforms from an HIV mAb. Fucose deficient mAbs exhibit enhanced antiviral activities. |
| Castilho A, Strasser R, Stadlmann J, Grass J, Jez J, Gattinger P et al. (2010) In *planta* protein sialylation through overexpression of the respective mammalian pathway. J Biol Chem. 285:15923-30. | 2010 | The reconstruction of the mammalian sialylation pathway in plants is shown. To achieve *in planta* sialylation six mammalian genes were transiently co-expressed in *N. benthamiana* |
| Castilho A, Gattinger P, Grass J, Jez J, Pabst M, Altmann F, et al (2011) N-glycosylation engineering of plants for the biosynthesis of glycoproteins with bisected and branched complex N-glycans. Glycobiology 21:813-23. | 2011 | By the transient overexpression of mammalian enzymes (i.e. GnTIII, GnTIV and GNTV) in *N.benthamiana* human like bisected and branched N-glycan structures were generated. The importance of sub Golgi targeting of the enzymes is demonstrated. |
| Zeitlin L, Pettitt J, Scully C, Bohorova N, Kim D, Pauly M, Ngo L, Steinkellner H, Whaley K, Olinger G Zeitlin L Design and testing of an Ebola virus post-exposure immunoprotectant: enhanced potency of a fucose free monoclonal antibody. PNAS | accepted | CHO produced and fucose free plant derived anti Ebola mAb 13F6 was compared in a lethal mouse virus challenge model. Plant derived 13F6 exhibited enhanced potency. |
